# The Nuclear Receptor DHR3 Modulates dS6 Kinase–Dependent Growth in *Drosophila*

**DOI:** 10.1371/journal.pgen.1000937

**Published:** 2010-05-06

**Authors:** Jacques Montagne, Caroline Lecerf, Jean-Philippe Parvy, Janis M. Bennion, Thomas Radimerski, Marie-Laure Ruhf, Frederic Zilbermann, Nicole Vouilloz, Hugo Stocker, Ernst Hafen, Sara C. Kozma, George Thomas

**Affiliations:** 1 Friedrich Miescher Institute for Biomedical Research, Basel, Switzerland; 2 Centre de Génétique Moléculaire, CNRS UPR 2167, Gif-sur-Yvette, France; 3 Université Paris-Sud, Orsay, France; 4 Université Pierre et Marie Curie-Paris 6, Paris, France; 5 Metabolic Diseases Institute, University of Cincinnati, Cincinnati, Ohio, United States of America; 6 ETH, Institut für Molekulare Systembiologie, Zürich, Switzerland; Harvard Medical School, Howard Hughes Medical Institute, United States of America

## Abstract

S6 kinases (S6Ks) act to integrate nutrient and insulin signaling pathways and, as such, function as positive effectors in cell growth and organismal development. However, they also have been shown to play a key role in limiting insulin signaling and in mediating the autophagic response. To identify novel regulators of S6K signaling, we have used a *Drosophila*-based, sensitized, gain-of-function genetic screen. Unexpectedly, one of the strongest enhancers to emerge from this screen was the nuclear receptor (NR), *Drosophila* hormone receptor 3 (DHR3), a critical constituent in the coordination of *Drosophila* metamorphosis. Here we demonstrate that DHR3, through dS6K, also acts to regulate cell-autonomous growth. Moreover, we show that the ligand-binding domain (LBD) of DHR3 is essential for mediating this response. Consistent with these findings, we have identified an endogenous DHR3 isoform that lacks the DBD. These results provide the first molecular link between the dS6K pathway, critical in controlling nutrient-dependent growth, and that of DHR3, a major mediator of ecdysone signaling, which, acting together, coordinate metamorphosis.

## Author Summary

In biological systems, the execution of morphogenic programs requires coordinated integration of the essential processes of growth, proliferation, and differentiation. Signaling networks embedded within these processes include the insulin and nutrient pathways required for cell growth and the steroid hormone-regulated pathways that control discrete developmental steps. Although these pathways are known to be integrated and coordinated, the molecular bridges that link them remain to be identified. Taking advantage of *Drosophila*, we performed a genetic screen for novel regulators of the dS6K, which previously has been identified as a key effector of cell growth downstream of insulin and nutrient signaling. Unexpectedly, we identified the nuclear receptor DHR3, a key regulator of morphogenesis, as a potent modulator of dS6K–mediated cell growth. Nuclear receptors typically comprise a DNA–binding domain and a regulatory ligand-binding domain. Here we show that a DHR3 isoform, devoid of the DNA–binding domain is sufficient to potentiate dS6K–mediated cell growth through its ligand-binding domain. We further demonstrate that, like dS6K, DHR3 regulates cell-autonomous growth. These data provide a unique molecular link between steroid-regulated development and nutrient-dependent growth.

## Introduction

During development, cell growth arrests when organs reach their appropriate size [1] such that differentiation acts to impede further growth. However, the growth-regulating module can be reactivated in specific cell types to maintain homeostasis in the adult. Moreover, pathological settings, such as cancer and obesity, can lead to aberrant activation of cell growth in a differentiated setting [2]. Despite this understanding, we have little knowledge of the molecular links that act to integrate differentiation programs with those that control growth. In identifying the underlying molecular mechanisms that regulate cell growth and differentiation in mammals, *Drosophila* genetics has proved a powerful tool. This is because many of the molecular components are evolutionarily conserved, as are the regulatory pathways in which they function [3]. In cell growth, such studies have been critical in revealing the central role of the Target of Rapamycin (TOR) as an effector of an insulin- and nutrient-signaling network that acts to maintain cell, tissue, and organismal homeostasis [4]. The value of *Drosophila* genetics in such studies was initially demonstrated in the identification of the genes responsible for Tuberous Sclerosis Complex, dTsc1 (hamartin), and dTsc2 (tuberin), as negative effectors of dTOR signaling [5]–[7] and subsequently the identification of their target, the small GTPase Ras homologue enriched in brain (dRheb) [8]–[10], a direct effector of TOR signaling [11].

In *Drosophila*, both the insulin-related peptides (Dilps), acting through the insulin receptor [12], and nutrients [13], such as amino acids acting through their cognate transporters [14], integrate at the level of dTOR to control cell growth [15], [16]. A key downstream effector of insulin- and nutrient-mediated dTOR-dependent growth is the *Drosophila* ribosomal protein S6 kinase (dS6K) [13], [17]. Although loss of dS6K largely results in late larval lethality, the few escapers that survive to adulthood are severely delayed in development and exhibit pronounced defects in cell size, with no effect on cell number [18]. Moreover, such mutants express elevated levels of Protein Kinase B (PKB) activity [17], which is mediated through a dS6K-negative feedback loop [15]. Although many of the effects of loss of dS6K appear to be controlled in a cell-autonomous manner [17]–[19], it is known that loss of the dS6K orthologue, S6K1, has humoral effects in the mouse [20]. Consistent with these findings, depletion of the amino acid transporter *slimfast* within the fat body (FB) reduces dS6K activity and causes a global growth defect similar to that seen in loss-of-dS6K mutants and nutritionally deprived *Drosophila* [14].

Following embryogenesis, *Drosophila* larvae, which are specialized in feeding and growth, increase their mass approximately 200 fold [21]. During this phase, endoreplicative tissues assume specific physiological functions, whereas the imaginal discs grow and proliferate [22]. At the termination of larval development, overall growth and feeding ceases. However, with the onset of metamorphosis, most of the endoreplicative organs are degraded, whereas the imaginal discs grow and differentiate into adult structures [23]. Metamorphosis is initiated by a peak in production of the steroid hormone ecdysone, which induces the activation of a cascade of nuclear receptors (NRs) [24] and the ensuing program of tissue remodeling. During metamorphosis, the degradation of the endoreplicative tissues, including the salivary gland and the midgut, is initiated by autophagy [25], a cellular process in which portions of cytoplasm are sequestered within double-membrane vesicles known as autophagosomes before delivery to lysosomes for degradation and recycling of cellular components [26]. Interestingly, although the dTOR signaling pathway acts as a negative effector of autophagy, there is evidence that dS6K promotes rather than suppresses this response [27], revealing a mutual dependency between these two pathways. Moreover, treatment with rapamycin, the inhibitor of dTOR/dS6K activation, blocks the production of ecdysone [28], which is mediated by prothoracicotropic hormone (PTTH) [29]. Although a connection between the signaling pathways induced by ecdysone and those induced by nutrients has not yet been formally established, earlier studies indicated that ecdysone antagonizes insulin and dTOR signaling [30]–[33]. However, recent findings demonstrate that during metamorphosis ecdysone also induces the fat body to produce Dilp6, which mediates the growth and proliferation of mitotic cells of the imaginal discs during the remodeling of tissues [23], [34].

In search of novel effectors of dS6K signaling, we have taken advantage of a sensitized phenotype, such that ectopic expression of dS6K within the developing dorsal wing compartment causes the wing to bend down [18]. This phenotype is characterized by an increase in the size of the dorsal wing blade, attributable to an increase in cell size, which is mediated by the level of dS6K activity [17], [19]. Using this sensitized phenotype in a genome-wide genetic screen, we have identified a number of potential effectors in dS6K signaling. Unexpectedly, one of the strongest amongst these was the NR DHR3, a critical signaling component in the coordination of *Drosophila* metamorphosis. Moreover, we show that the ligand-binding domain of DHR3 is essential in modulating dS6K-regulated cell growth, which led us to the identification of a novel isoform of DHR3, devoid of the DNA-binding domain.

## Results

### A screen for dS6K modulators

**Figure 1:**
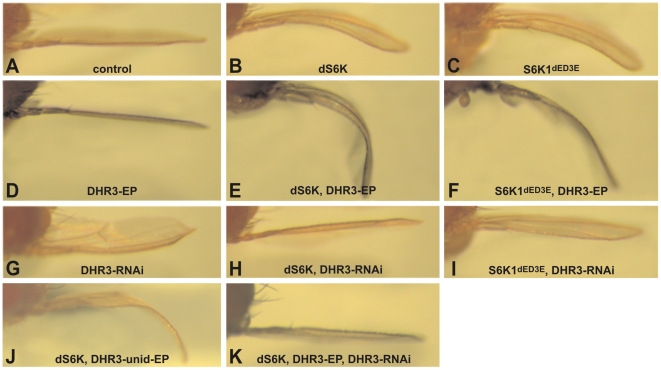
DHR3 is a specific dS6K interacter. Induction of UAS constructs and EPs by the *ap* promoter directed Gal4 expression within the dorsal compartment of the wing imaginal disc: (A) ap-Gal4 control; (B, E, H, J, K) UAS-dS6K; (C, F, I) UAS-S6K1^dE/D3E^; (D, E, F) DHR3-EP; (G, H, I, K) UAS-DHR3-RNAi and (J) unidirectional DHR3-EP. The bending down of the wing indicates a slight overgrowth of the dorsal compartment, whereas a bending up reveals a slight growth deficit of this compartment.

Imaginal discs are subdivided into compartments, with each constituting an individual growth unit that differentiates into an adult structure during metamorphosis [22]. In this context, ectopic expression of dS6K within the developing dorsal wing compartment, using the *apterous*-Gal4 (*ap*-Gal4) driver, induces a moderate overgrowth in this unit [18] and a bending-down of the adult wing (Figure 1A and 1B). Consistent with PDK1 being the mammalian S6K1 activation-loop kinase [35], [36], we have previously demonstrated an enhanced bending-down of the adult wing by co-expression of the *Drosophila* PDK1 (dPDK1), whereas expression of dPDK1 alone had no effect on this phenotype [17]. Likewise, the expression of particular phosphorylation-site mutants of dS6K that would be predicted to increase or decrease the activation state of dS6K, enhances or suppresses this phenotype, respectively [19]. These findings demonstrate that the bent-down wing phenotype varies according to dS6K activation status, and prompted us to use this sensitized phenotype in a gain-of-function genetic screen to identify novel modulators of dS6K activity. We also found that ectopic expression of an active form of the mammalian S6 Kinase 1, S6K1^dE/D3E^ [37], induced a bent-down wing phenotype equivalent to that induced by dS6K (compare Figure 1B and 1C). Like dS6K, co-expression of S6K1^dE/D3E^ and dPDK1 led to an enhancement of the bent-down wing phenotype, but not to the extent observed with dS6K (data not shown). We reasoned that this differential phenotype may represent dPDK1 specificity for dS6K, a *bona fide* substrate, unlike S6K1^dE/D3E^ [17], [38], and have utilized this differential effect to increase the selectivity of the gain-of-function screen (see below).

In the screen, approximately five thousand Enhancer-Promoter (EP) bi-directional insertions were co-induced with dS6K in the developing dorsal wing compartment [39] and then scored for either enhancement or suppression of the dS6K-dependent bent-down wing phenotype. Approximately 1000 of the EP lines either acting as suppressors (∼90%) or enhancers (∼10%), were further analyzed for their effects on the bent-down wing phenotype when induced alone or with the ap-Gal4 driver. In this way, nonspecific effectors, which alone could induce the bent-wing phenotype, were eliminated (data not shown), allowing us to narrow down the potential candidates to 220 lines. These were then tested in the tertiary screen in combination with either dS6K or the active S6K1^dE/D3E^, such that 19 suppressor and 76 enhancer lines were retained (Table S1), 71 of which were localized by reverse PCR mapping. We focused on the 76 enhancer lines, which were largely confirmed through the three screening steps. Of these, 19 were not considered because they interfered with wing development (Table S1), including perturbation of vein formation, compartment adhesion (Blister phenotype in Table S1), or the bending down of the wing along the anterior-posterior axis (Figure S1). Among the 57 enhancer lines selected through this process, the candidate list was further narrowed to the strongest 21 enhancers (Table S1), of which 18 induced a more severe phenotype with dS6K than with S6K1^dE/D3E^.

### DHR3, a genetic modulator of dS6K–dependent growth

That the 18 strong enhancers represented authentic dS6K interacters was supported by the finding that 9 of the enhancers were localized as independent insertions in the dPDK1 locus (Table S1). Genomic mapping of the additional strong enhancers led to the identification of 5 novel loci. Of these, we focused our attention on EP lines EP12.218 and EP23.014 (Figure S2), which were inserted in the DHR3 locus, coding for a nuclear receptor (CG33183), and are collectively referred to as DHR3-EP. Induction of either of these EP lines alone, using the *ap*-Gal4 driver, was without visible effect on wing development (Figure 1D and data not shown); whereas, in combination with dS6K or S6K1^dE/D3E^ both EP lines induced a strong enhancement of the bent-down wing phenotype (compare Figure 1B and 1C with Figure 1E and 1F, respectively, and data not shown). As the EP element employed in the screen contained two UAS promoters to direct transcription in opposite directions [39], the DHR3-EP could, theoretically, induce transcription of either *DHR3* or the histidine-decarboxylase gene (CG3454). As the UAS promoter driving the latter gene was flanked by loxp sequences, induction of Cre recombinase was used to excise this promoter in the EP23.014 line. The resulting unidirectional EP line retained the ability to enhance the dS6K-induced bent-down wing phenotype (Figure 1J) arguing that the EPs mediate their effects through DHR3. Using this same approach, we determined that the four additional loci most likely regulate the expression of *rab40* (CG1900), involved in vesicle trafficking; *peste*, encoding a scavenger protein (CG7228); *orb2*, a Cytoplasmic-Polyadenylation-Element-Binding (CPEB) protein (CG5735); and *hephaestus*, a Polypyrimidine-Track-Binding (PTB) protein (CG31000) (Table S1).

As both DHR3-EP elements are inserted within the first intron of the DHR3 gene (see below), it is most likely that the enhanced bent-down wing phenotype results from either inhibiting or increasing the expression of DHR3. We therefore generated inducible UAS-RNA-interference lines (DHR3-RNAi) to specifically reduce DHR3 expression. Induction of the DHR3-RNAi alone by *ap*-Gal4 caused a bending up of the wing (Figure 1G), indicating that normal growth of the dorsal wing blade is restricted when DHR3 expression is suppressed. Further supporting this observation, when co-induced in the dorsal wing compartment with either dS6K alone or in combination with DHR3-EP, the DHR3-RNAi completely suppressed the bent-down wing phenotype in both settings (Figure 1H and 1K). These findings indicate that the positive genetic interaction observed with dS6K is due to increased expression of the DHR3 gene product. Although we found that co-expression of DHR3-EP and S6K1^dE/D3E^ enhanced the bent-down wing phenotype (Figure 1C and 1F), this was to a lesser degree than when co-expressed with dS6K (compare Figure 1E and 1F), suggesting that DHR3, like dPDK1, acts specifically on dS6K signaling. Consistent with this interpretation, DHR3-RNAi suppressed the bent-down wing phenotype induced by dS6K more strongly than that of S6K1^dE/D3E^ (compare Figure 1H and 1I). Taken together, these differential effects indicate that S6K1^dE/D3E^ is less sensitive than dS6K to relative changes in the dosage of DHR3 and favor a specific role for DHR3 in dS6K-dependent growth.

### DHR3–EP reverses the inhibitory effects of dTsc1/2, but not dPTEN, on growth

**Figure 2:**
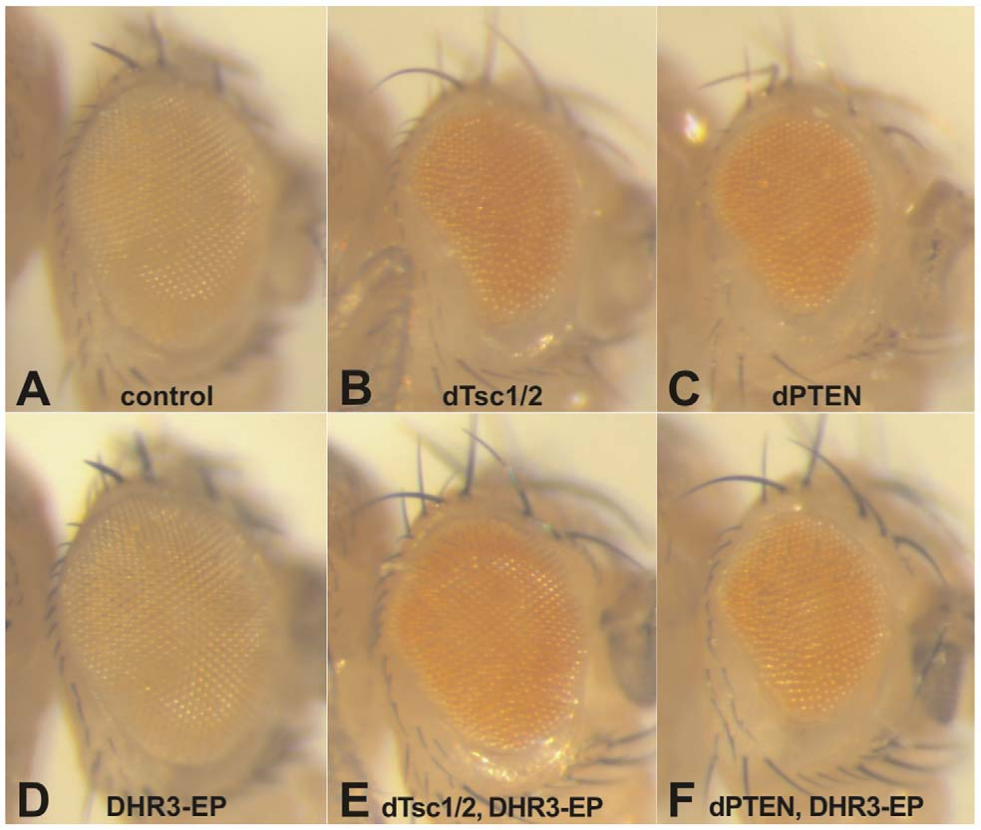
DHR3–EP specifically counteracts Tsc-dependent growth suppression. Induction of UAS constructs and DHR3-EP by the eyeless promoter-directed Gal4 expression in the developing eye: (A) eye-Gal4 control; (B, D, E) DHR3-EP; (C, D) UAS-dTsc1 and UAS-dTsc2; (E, F) UAS-dPTEN.

Recently, it has been suggested that the nutrient-effector arm of the TOR signaling pathway may have been integrated with that of the insulin-PI3K pathway following the rise of multicellular organisms [40]. Although it is clear that the nutrient and insulin pathways are also integrated in *Drosophila*, it is less clear where the point of integration resides [15], [17]. In part, this lack of clarity resides in the finding that depletion dTsc1/2, but not dPTEN, leads to dS6K activation, and that the overgrowth phenotype caused by loss of dTsc1/2, but not of dPTEN, is abolished by loss of dS6K [17]. Consistent with these findings, when either tumor suppressor is ectopically expressed in the developing eye, they suppress growth of this compartment, with co-expression of dS6K counteracting only the effects of dTsc1/2, but not of dPTEN (data not shown). This difference allows us to test whether DHR3-EP is acting exclusively on the dTsc1/2 growth response. As stated above, ectopic expression of either dTsc1/2 or dPTEN suppressed the growth of the developing eye (compare Figure 2A–2C). In contrast, ectopic expression of DHR3-EP had no apparent impact on eye development (compare Figure 2A and 2D), similar to what was observed in the wing (Figure 1D). However, ectopic expression of DHR3-EP, combined with either dTsc1/2 or dPTEN, largely counteracted the growth-suppressive effects due to dTsc1/2, but not of dPTEN (compare Figure 2E and 2B, and Figure 2F with 2C). These results support the notion that DHR3 acts to promote dS6K signaling.

**Figure 3:**
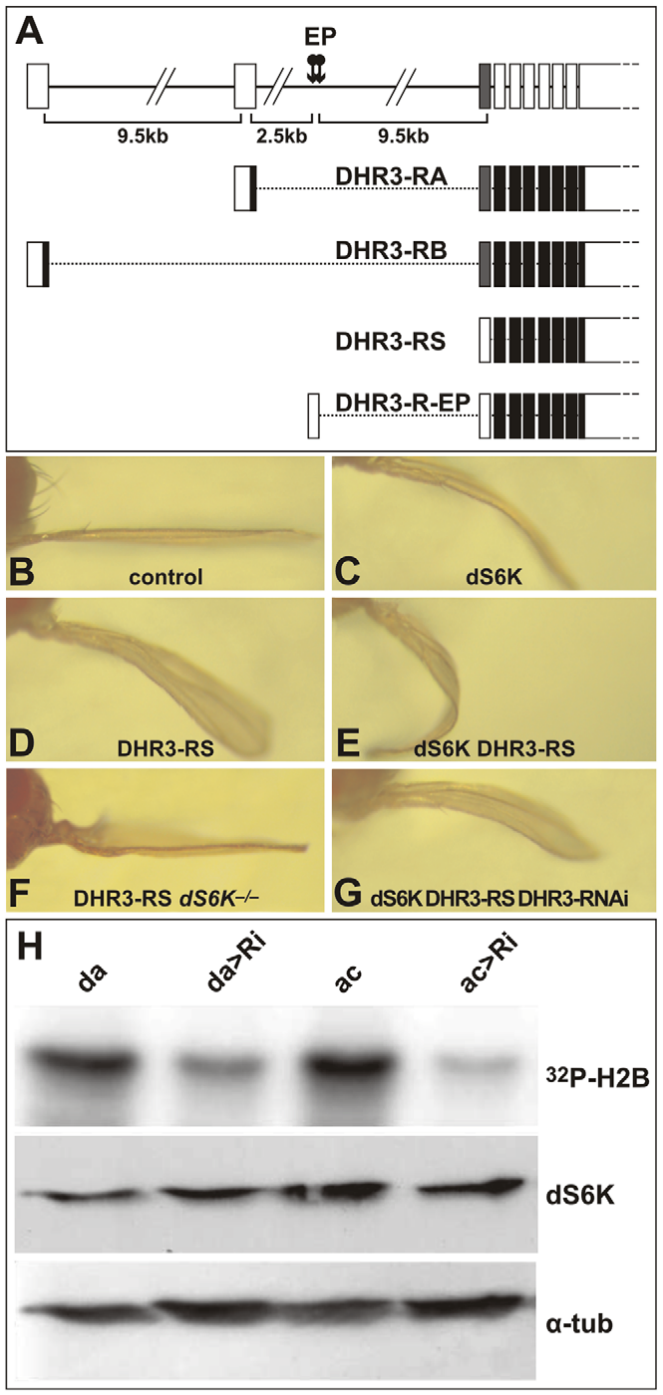
DHR3 regulates dS6K activity. (A) The DHR3 locus is schematized at the top with the 2 EP insertions indicated (arrows). The RA and RB transcripts encode a DBD sequence (indicated by the grey box). RS is a putative novel transcript whose first AUG is located beyond the DBD-coding sequences. (B–G) Adult wings in which the *ap*-Gal4 induces expression of the following UAS constructs: (B) *ap*-Gal4 control; (C) UAS-dS6K; (D) UAS-DHR3-RS; (E) both UAS-dS6K and UAS-DHR3-RS; (F) UAS-DHR3-RS in a *dS6K^l-1^* mutant escaper; (G) UAS-dS6K, UAS-DHR3-RS, and UAS-DHR3-RNAi together. (H) Upper panel, H2B substrate phosphorylation by dS6K from larval protein extracts. Ubiquitous induction of UAS-DHR3 RNAi, using either a daughterless-Gal4 (da>Ri) or an actin-Gal4 (ac>Ri) driver, provoked a drop in dS6K activity, as compared with control drivers alone (da, ac). Middle and lower panels are western blots detecting dS6K and α-tubulin, respectively.

DHR3 is known to play a central role in coordinating metamorphosis [41], [42]; however, when DHR3-RNAi was expressed in the dorsal wing compartment it led to a decrease in the size of the dorsal wing blade, causing the wing to bend upwards, with no obvious detrimental effect on the differentiation of the wing (Figure 1G). In agreement with this finding, DHR3-EP suppressed the growth defect induced by overexpression of dTsc1/2 in the eye, without altering differentiation of this organ (Figure 2E). These findings were unexpected as they suggest that DHR3 is not only involved in fate decisions associated with differentiation, but that it may also play an integrative role in controlling cell growth. Both EP elements were inserted within the large first intron of DHR3, and failed to complement previously reported DHR3 mutants (Figure 3A and Figure S2; Table S2). However, in contrast to these previously described DHR3 mutants, which are lethal during early development, homozygous and trans-heterozygous DHR3-EP insertions are semilethal (data not shown), indicating that they represent hypomorphic DHR3 mutants. The few larvae that underwent metamorphosis were delayed (data not shown) and exhibited a significant reduction in body weight (Figure S3). The adult escapers emerged with an approximate 2-day delay, and displayed female sterility. A reduction in body weight and developmental delay have been reported for a number of other mutants that affect growth, further supporting a role for DHR3 in controlling this process [18], [43]. Consistent with this observation, we also found that ubiquitous suppression of DHR3 by RNAi provoked larval death, but also provoked a significant developmental delay (data not shown). Taken together, these findings imply that DHR3 has a distinct function in controlling cell growth, potentially through dS6K.

### The DHR3 protein that interacts with dSK6 lacks a DBD

DHR3 is a NR that classically comprises an amino-terminal DNA-binding-domain (DBD) and a carboxyl-terminal ligand-binding domain (LBD), separated by a linker domain [44]. The FlyBase Consortium [45] first reported two potential transcripts for DHR3 termed RA and RB (“R” stands for RNA, whereas “P” denotes the corresponding protein) (Figure 3A), though, more recently, two additional transcripts, RC and RD, have been listed. All reported DHR3 polypeptides are translated from AUGs located at specific alternative upstream first exons (Figure 3A and data not shown). To identify the DHR3 gene product responsible for the genetic interaction with dS6K, RACE (rapid amplification of cDNA ends)-PCR has been performed using wild-type and DHR3-EP larvae ubiquitously induced by a *daughterless*-Gal4 driver (*da*-Gal4). The transcript identified for the latter was a splice variant extending from the EP to the DHR3 second exon, and lacking an AUG initiator codon upstream of the sequences encoding the DBD (R-EP in Figure 3A and Figure S2). In addition to previously described mRNAs, RACE-PCR experiments using wild-type larvae revealed a novel DHR3 transcript lacking a first alternative exon (RS, where S stands for short, Figure 3A and Figure S2). This transcript would be predicted to encode a DHR3-PS protein that is devoid of the DBD, as the most proximal AUG is located beyond the DBD-coding sequence (Figure 3A and Figure S2). The functional existence of a DHR3 isotype lacking the DBD is supported by the chimeric EP/DHR3 transcripts. To determine which DHR3 isotype was responsible for the genetic interaction with dS6K, three UAS constructs were generated, two of which corresponded to the RA and RB transcripts described above. The third UAS construct, DHR3-RS, lacked an upstream translational initiator codon, but retained an AUG, to potentially allow translation of the PS variant (Figure 3A and Figure S2). When induced by the *ap*-Gal4 driver, both RA and RB led to lethality (data not shown). This lethality was most likely due to expression in organs other than the wing, as the *apterous* promoter is known to be active in several tissues, including some embryonic neurons [46]. Conversely, induction of DHR3-RS with the *ap*-Gal4 driver was not lethal and phenocopied the enhancement of the dS6K wing phenotype observed with DHR3-R-EP (compare Figure 3C and 3E with Figure 1B and 1E, respectively). Moreover, co-induction of DHR3-RNAi suppressed this phenotype (compare Figure 3E and 3G), as it did when co-expressed with dS6K and the DHR3-R-EP (Figure 1K). Interestingly, induction of DHR3-RS alone was sufficient to induce a bent-down wing (compare Figure 3B and 3D), and this phenotype was largely reverted by co-induction of DHR3-RNAi (data not shown). Hence, expression of the DHR3 gene product lacking its DBD alone is sufficient to induce growth of imaginal discs and can further cooperate with dS6K in this process.

### DHR3 regulates dS6K activity

The ability of *ap*-Gal4-driven DHR3-RS alone to induce the bent-down wing phenotype (Figure 3D), as compared with DHR3-EP (Figure 1D), could be explained by higher expression levels of DHR3-PS (see below). Combined with data in Figure 1, these data also suggest that DHR3-RS–driven growth relies on dS6K. To test this possibility, we induced *ap*-Gal4-driven DHR3-RS in the *dS6K^l-1^* null-mutant, of which a small number survive to adulthood [18]. In this genetic background, a clear suppression of the bent-down wing phenotype was observed (compare Figure 3F and 3D), indicating that overgrowth induced by DHR3-RS is dependent on the presence of dS6K. The genetic interactions between DHR3 and dS6K raised the possibility that DHR3 might control either dS6K levels or activity. To discriminate between these two possibilities, ubiquitous expression of DHR3-RNAi was induced by a da-Gal4 or actin-Gal4 driver, and both the level and the activity of dS6K were monitored in larval extracts. With either driver, RNAi-induced DHR3 suppression led to a strong reduction in dS6K activity, as measured by histone 2B (H2B) phosphorylation (Figure 3H) or dTORC1-dependent phosphorylation of dS6K1 T398 [47](Figure S4). Under these conditions there was no effect on dS6K protein levels (Figure 3H and Figure S4). Importantly, RNAi-induced DHR3 suppression also suppressed dTORC1-dependent phosphorylation of d4E-BP T37/T46 (Figure S4), the inhibitor of the translation initiation factor d4E [48]. The results indicate that DHR3 is required during larval development to maintain full dS6K activity, potentially acting through dTORC1.

### A DBD–lacking DHR3 protein

**Figure 4:**
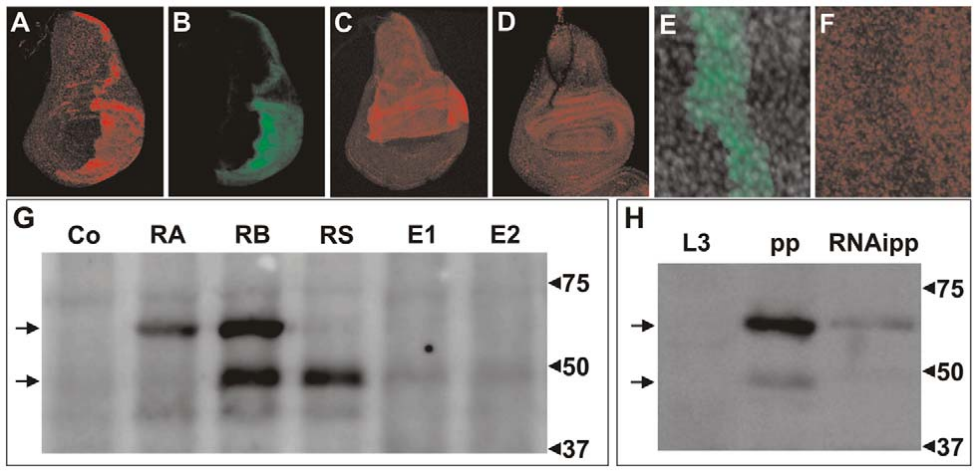
Immunodetection of DHR3 proteins. (A–F) Wing imaginal discs stained with: (A, C, D, F) antibody to DHR3, and (B, E) antibody to GFP. (A, B) Overexpression of DHR3-RS and USA-GFP in the posterior compartment was induced using an engrailed-Gal4 driver. (C) and (D) *ap*-Gal4 drives overexpression of DHR3-RS and DHR3-EP, respectively. (E, F) Flip-out clone (induced 3 days prior to dissection) in a prepupal wing imaginal disc expressing UAS-DHR3-RNAi and UAS-GFP. (E) Decrease in DHR3 staining in a flip-out clone labeled by GFP (F), indicating that DHR3 is expressed in this tissue. (G) Western blot analysis of DHR3 in larval protein extracts from control (Co) and heat shock-induced UAS lines expressing DHR3-RA (RA), DHR3-RB (RB), and DHR3-RS (RS) transcripts, or the DHR3-EPs (E1 and E2). (H) Western blot of the endogenous DHR3 protein in late third-instar larvae (L3), prepupae (pp), and prepupae expressing an RNAi to DHR3 (RNAipp). In (G, H), the arrows at the left indicate the position of the DBD-containing (high) and DBD-lacking (low) DHR3 proteins.

To determine whether the endogenous isoform DHR3-PS, lacking the DBD, is expressed in vivo, a rabbit antiserum to DHR3 was produced using peptides that correspond to sequences downstream of the first AUG following the DBD coding sequence (Figure S2). Expression of the UAS–DHR3-RS (Figure 3A), was induced in the posterior wing-disc compartment using the engrailed-Gal4 (en-Gal4) driver. This line also harbored a UAS-GFP, activated by the en-Gal4 driver leading to the production of GFP, which allowed for double immunostaining. The results of this experiment revealed co-localization of GFP and DHR3-PS expressions (Figure 4A and 4B). Likewise, when induced by the *ap*-Gal4 driver, both the DHR3-RS and the DHR3-EP lines exhibited increased immunostaining within the dorsal wing-disc compartment, which was much stronger for DHR3-RS than for DHR3-EP (compare Figure 4C and 4D). Because DHR3-RS, but not DHR3-EP, provoked the bent-down wing phenotype when induced alone by *ap*-Gal4 (compare Figure 1D with Figure 3D), these results are consistent with the ability of DHR3-RS to induce growth in a dosage-dependent manner. To determine whether we could also detect endogenous DHR3, flip-out clones directing DHR3-RNAi expression were generated, and a UAS-GFP was used to positively label these clones [49]. The staining observed in prepupal discs was strongly reduced in flip-out clones (Figure 4E and 4F), with remnant staining most likely reflecting incomplete depletion of DHR3 expression. Clones displaying a decrease in specific staining could be detected in all imaginal discs from prepupae (data not shown), indicating that DHR3 is widely represented at this stage of development. In addition, weak staining could be detected in both the imaginal discs and the fat body from mid-third-instar larvae (data not shown), suggesting the presence of low levels of DHR3 at this stage. Thus, endogenous DHR3 is detectable in prepupae, but also likely present at low levels in larval tissues.

To analyze the distinct DHR3 polypeptides by western blotting, expression of the UAS–cDNAs, DHR3-RA, RB, RS, or R-EP (Figure 3A), were induced by a one-hour heat-shock treatment using the heat-shock-Gal4 driver (HS-Gal4). Because larvae expressing the DBD-containing DHR3 isotypes died within a day following heat shock, larval extracts were prepared four hours after heat shock and analyzed by western blotting. Larvae expressing the DBD-containing DHR3 variants (RA and RB) displayed distinct protein patterns. DHR3-RA produced a single protein that migrated at the expected molecular weight for PA (Figure 4G, lane RA). Similarly, DHR3-RB produced a band migrating at a molecular weight very similar to that of PA, which most likely represented PB (Figure 4G, lane RB). Unexpectedly, DHR3-RB also produced a second polypeptide migrating at a significantly smaller molecular weight (Figure 4G lane RB). Consistent with this latter polypeptide representing the DHR3 variant lacking the DBD, the DHR3-RS and the two DHR3-EP lines (Figure 4G, lanes RS, E1, and E2) produced a protein that migrated at the same position as the smaller polypeptide produced by DHR3-RB (Figure 4G, lane RB). According to the immunostaining (Figure 4C and 4D), the DHR3-RS line expressed significantly more protein than the two DHR3-EP lines (compare Figure 4G, lanes RS, E1, and E2). As DHR3 has been reported to be highly expressed at the onset of metamorphosis in response to ecdysone signaling [50], we monitored its expression pattern by western blot analysis in third-instar larvae and during pupariation. Neither the long nor the short forms of DHR3 could be observed in late third-instar larvae, but both were clearly detectable in prepupae (Figure 4H). That these two bands represent DHR3 was shown by their reduced expression levels in prepupae expressing the DHR3-RNAi using a *da*-Gal4 driver (Figure 4H). The smaller protein was most likely produced from the DHR3-RB transcript or, alternatively, from the RS messenger species devoid of an upstream AUG (Figure 3A). These data are consistent with the surge of DHR3 expression during pupariation.

### The ligand-binding domain of DHR3 is required for cell-autonomous growth

**Figure 5:**
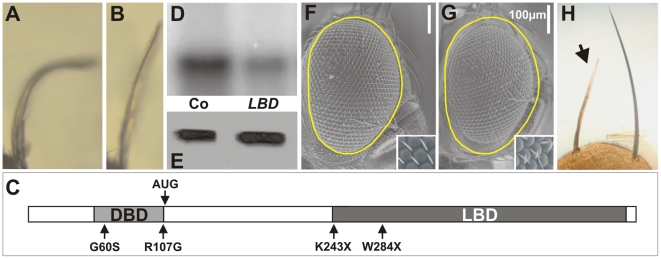
Mutations in the LBD of DHR3. (A, B) The *ap*-Gal4 driver induces dS6K in combination with either (A) the DHR3-EP transgene or (B) the EP with the EMS DHR3 mutation. (C) Structure of the full-length DHR3-PA protein showing previously described DBD mutants (G60S, R107G) and the EMS mutants that lack the LBD (K243X, W284X); the light and dark grey boxes represent the DBD and the LBD respectively. (D) dS6K activity in larval protein extracts measured by H2B target phosphorylation and (E) corresponding western blot of dS6K in the trans-heterozygote LBD-mutant combination (LBD), as compared to heterozygous larvae (Co). (F, G) The eyeless promoter directs the flipase recombinase during eye development. Flipase allows recombination on the right arm of the second chromosome and eventually leads to an adult eye (F) that is homozygous for the wild-type allele of DHR3. (G) Homozygosity for a *DHR3^K243X^* mutation results in a decrease in the size of the adult eye. A dotted yellow line surrounding the control eye in (F) has been copied and pasted on the mutant eyes (G); insets are higher magnification images of ommatidia showing misorientation of bristles. (H) At the scutellum, a *y*-marked homozygous *DHR3^K243X^* mutant bristle (arrow), recognizable by its light color, is smaller than the symmetrical neighboring bristles.

To gain further insight into the protein domain of DHR3 required for the dS6K-dependent growth function, an EMS revertant screen was performed. DHR3-EP males were fed EMS and crossed to females bearing *ap*-Gal4–induced dS6K. Approximately 50,000 offspring were screened to establish 8 lines that had clearly lost the ability to cooperate with dS6K in producing the bent-down wing phenotype (compare Figure 5A and 5B). After remobilization of the EP-element, only two lines displayed homozygous lethality and did not complement previously described DHR3 mutants (Table S2). These two lines contained stop codons at positions 243 and 284 of the DHR3-PA reading frame, respectively, and are referred to as *DHR3^K243X^* and *DHR3^W284X^* (Figure 5C and Figure S2). Remobilization of the EP element may provoke imprecise excisions, creating putative deficiencies within the DHR3 locus. Hence, several lines for each DHR3 mutation were generated from independent remobilization events. Eight and ten independent lines for *DHR3^K243X^* and *DHR3^W284X^*, respectively, were used to further investigate the function of the DHR3 LBD. All were homozygous lethal, failed to complement one another, and neither complemented the previously described *DHR3^G60S^* and *DHR3^R107G^* mutants [51] that affect the DBD (Table S2). Almost all of these mutant combinations died as embryos indicating that the LBD is required for the transcriptional function of DHR3. However, it was possible to identify a few *DHR3^K243X^*/*DHR3^W284X^* mutants that survived to the second larval instar. These larvae were then used to perform kinase assays for dS6K. Consistent with the results of assays using DHR3-RNAi extracts (Figure 3H), a significant drop in dS6K activity, but not expression, was observed in larval extracts prepared from trans-heterozygous *DHR3^K243X^*/*DHR3^W284X^* mutants (Figure 5D and 5E).

To examine the LBD mutants with respect to cell-autonomous growth, both lines devoid of the EP-element were fused to an FRT, and using the flipase recombinase, analyzed in specific tissues of the adult [52]. We first investigated the FRT-associated mutations in the eye disc of heterozygous DHR3 mutant flies by using the *eyeless* promoter to drive flipase during eye development [53]. As the FRT chromosome arm carrying a wild-type DHR3 copy also contained a homozygous cell-lethal *Minute* mutation (*M(2)53*), the recombined sister cells, which were wild type for DHR3, were eliminated during development. This led to adult eyes that were largely made up of homozygous DHR3 mutant cells. With either the *DHR3^K243X^* or *DHR3^W284X^* mutation, a significant reduction in eye size was observed (Figure 5F and 5G, and data not shown), demonstrating that DHR3 controls growth in a compartment-autonomous manner. The flipase recombinase was also induced by heat shock, and adult homozygous DHR3 mutant clones were followed by their yellow marker. At the scutellum (posterior part of the dorsal thorax), DHR3 mutant *yellow* bristles were easily distinguishable from their neighbors and were significantly reduced in size (Figure 5H). Thus, mutations in the DHR3 ligand-binding domain appear to have significant effects on growth, independent of differentiation.

**Figure 6:**
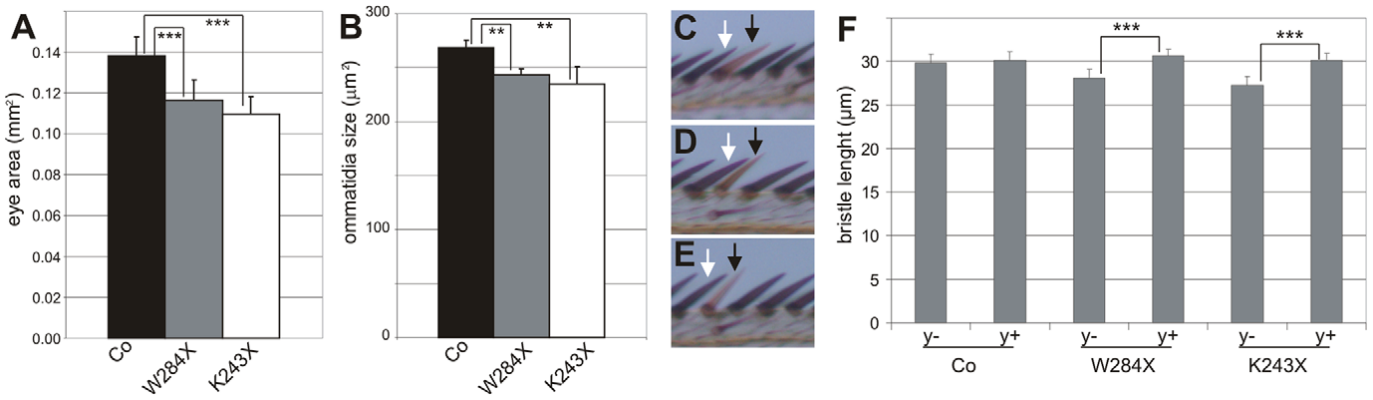
Growth defect in DHR3 LBD mutant cells. (A) Normalized eye size (mm^2^) of control (black bar; n = 20), *DHR3^W284X^* (grey bar; n = 18) and *DHR3^K243X^* (white bar; n = 18) mutants. As compared to control recombined eyes (Co), *DHR3^W284X^* and *DHR3^K243X^* mutant eyes exhibit a highly significant size reduction (***P<0.001). (B) Ommatidia size of control (black bar), *DHR3^W284X^* (grey bar), and *DHR3^K243X^* (white bar) mutants. As compared to control recombined eyes (Co), *DHR3^W284X^* and *DHR3^K243X^* mutant ommatidia exhibit a significant size reduction (**P<0.01). (C–E) Comparison of *y*-marked homozygous clonal bristles (black arrows) to their neighboring heterozygous bristles (white arrows) at the wing margin. Note that control clonal bristles are of normal size and orientation (C), whereas those of *DHR3^W284X^* (D) and *DHR3^K243X^* (E) are reduced in size and are misoriented. (F) Size measurements (µm) of the length of homozygous clones (*y−*) and their neighboring heterozygous (*y+*) bristles (n = 51 for each bar). The size reduction of clonal versus neighbor bristles is highly significant for *DHR3^K243X^* (***P<0.001) and *DHR3^W284X^* (***P<0.001) mutants, but not for the control (Co) bristles (P = 0.028).

To evaluate the growth defects due to DHR3 LBD mutation, statistical analyses were performed on the size of eyes and ommatidia as well as bristle length. Homozygous DHR3-mutant eyes were generated in a trans-heterozygous *M(2)53/DHR3^−^* mutant background, which produces variation in the body size of adult flies (data not shown). Therefore, the areas of the homozygous eyes were normalized to the areas of the corresponding heterozygous thoraces. As compared to control recombined eyes, the homozygous *DHR3^K243X^* and *DHR3^W284X^* mutant eyes exhibited a significant reduction in surface area (Figure 6A). The surface area of ommatidia from scanning electron micrographs of flies of equivalent size was also determined. Notably, the reduction in ommatidia area (Figure 6B) was not as strong as for the surface of the entire eye, indicating that the number of ommatidia was also affected. To precisely measure the effect on cell growth, bristle length was analyzed at the edge of the wing margin, as the shaft of each bristle corresponds to a single cell. Comparison of homozygous clonal bristles to the neighboring control bristles (Figure 6C–6E) revealed that the length of homozygous *yellow*-marked bristles was unaffected (Figure 6C and 6F), indicating that, in this setting, the *yellow* marker is appropriate to monitor cell-autonomous growth. In contrast, there was a significant reduction in the size of both *DHR3^K243X^* and *DHR3^W284X^* homozygous mutant bristles, as compared to the neighboring control bristles (Figure 6D–6F) indicating that the LBD of DHR3 is required to sustain cell-autonomous growth. The DHR3 homozygous mutant bristles were affected also in their orientation, as compared with the surrounding bristles (Figure 6D and 6E). Misorientation was also observed for the ommatidia-associated bristles in homozygous DHR3-LBD mutant eyes (insets in Figure 5F and 5G), potentially reflecting one of the pleiotropic functions of DHR3. Taken together, our findings demonstrate that, in addition to a role in coordinating the onset of metamorphosis, DHR3 also acts in a cell-autonomous manner to control cell growth.

## Discussion

By using *Drosophila* genetics and a gain-of-function strategy, we identified the NR, DHR3, as an enhancer of a dS6K-regulated growth phenotype. This effect can be mediated by an isoform of DHR3 lacking the DBD. Moreover, using a revertant screening strategy, we have generated LBD-specific DHR3 mutants and demonstrated that the LBD of DHR3 is necessary to maintain normal growth and dS6K activity. In contrast to the role DHR3 plays in transcriptional regulation affecting the onset of metamorphosis [41], [42], our studies indicate that it also plays a role in regulating cell-autonomous growth. These effects are most likely mediated through dS6K, as the ability of ectopically expressed DHR3-RS to drive growth in the dorsal wing blade is blunted in *Drosophila* deficient for dS6K. Consistent with these findings, we have previously demonstrated that dS6K also controls cell growth in a cell-autonomous manner [18]. However, the effect on cell size is more pronounced in dS6K mutants [18] than in the DHR3-mutant clones described here. This may reflect the fact that dS6K activity is blunted, but not abolished, in DHR3 LBD-mutant larvae. Compatible with this hypothesis, we previously found that in a dS6K P-element–induced mutant (P{PZ}S6K[07084]) we could not detect dS6K protein (unpublished results); however, this mutation induced a much less severe phenotype as compared with the *dS6K^l-1^* null mutation [18]. In homozygous DHR3 mutant eyes both the size and the number of ommatidia were decreased, whereas in dS6K mutant flies the size reduction of the eye was only due to a decrease in ommatidia size but not number [18]. This difference might be attributed to the experimental settings. In the current study, DHR3 mutant eyes were generated by mitotic recombination in a heterozygous *Minute* background, whose developmental delay is less than two days. In contrast, the size and number of ommatidia in dS6K mutant eyes were measured in homozygous mutant flies that exhibit a five-day delay at eclosion. The longer time for the latter to emerge as adults allows additional cell divisions to proceed, leading to a higher number of ommatidia [54].

Previous studies demonstrated that DHR3 participates in a hierarchal regulatory circuit in response to ecdysone signaling [41], [55], but also acts in a negative feedback loop to repress ecdysone receptor-mediated signaling [42]. Prothoracic gland production of ecdysone is mediated by the brain neuropeptide prothoracicotropic hormone (PTTH) [56]. Recent studies in *Drosophila* have shown that genetic ablation of PTTH-producing neurons induces a delay in larval development and results in larger adult flies as a direct consequence of reduced levels of ecdysone [29]. Interestingly, in the tobacco hornworm, *Manduca sexta*, PTTH-induced ecdysone production is paralleled by the phosphorylation of the *Manduca* orthologue of *Drosophila* ribosomal protein S6 [28]. Moreover, this process is sensitive to rapamycin [28] and we have observed a burst of dS6K activity at early pupation (unpublished data). As the body size of the adult fly appears to be determined by growth regulators, including dS6K, as well as by hormones that control the timing of developmental windows, such as PTTH, our results suggest that the DHR3/dS6K regulatory module acts to integrate these two processes.

The studies presented here support the existence of a novel DHR3 polypeptide devoid of a DBD, DHR3-PS. Nonetheless, although DHR3-PS is sufficient to potentiate a dS6K-dependent growth phenotype, we can not exclude that the other DBD-containing DHR3 isoforms also contribute to dS6K activation. In general, DHR3, like other NRs, is a transcription factor composed of four elements: a modulator domain, the DBD, the hinge region, and the LBD [57]. The DBD of NRs typically consists of two zinc fingers, with the first being critical for conferring DNA-binding specificity [58]. Like DHR3-PS, NRs lacking a DBD have been previously reported. Notably, in *Drosophila*, the NR E75B, a DHR3 partner, lacks one of the 2 zinc fingers that is required to form a functional DBD [59]. However, E75B, through its ability to interact with DHR3, modulates DHR3 transcriptional activity in a gas-responsive manner [60]. Like the putative DHR3-PS, the NR short heterodimer partner (SHP) in mammals is also devoid of DBD, but, as with E75B, it interacts with other NRs to modulate their transcriptional activity [61]. It is unlikely that DHR3-PS behaves as a dominant-interfering effector of full-length DHR3 as ectopic DHR3-PS expression induces growth, whereas DHR3-RNAi inhibits growth. However, DHR3 also heterodimerizes with two NRs: E75 and the ecdysone receptor [41], [42]. Thus, in the case of E75, ectopically expressed DHR3-PS may act to decrease the levels of free E75, leaving full-length DHR3 free to increase the transcription of target genes. In contrast, DHR3-PS binding to the ecdysone receptor could counteract the negative growth regulation mediated by ecdysone signaling [31]. However, it should be noted that the negative effects of ecdysone are humoral [33] and mediated by dFOXO-inactivation within the fat body [31], whereas, as we have shown here, DHR3 regulates growth in a cell-autonomous manner. Moreover, dFOXO subcellular distribution was not altered in DHR3 mutant clones in third instar wing imaginal discs (data not shown), indicating that the DHR3 cell-autonomous effect on cell growth is not mediated by the PKB/dFOXO signaling.

In contrast to acting as a dominant-interfering isoform, the results presented here also suggest that DHR3 activates dS6K through a non-genomic mechanism, an effect of NRs that does not require the DBD function. Such a model is supported by NR responses whose kinetics are too rapid to be explained by de novo transcription and translation of a gene product [62]. Indeed, nongenomic effects typically occur within minutes following addition of the cognate ligand and are resistant to transcriptional inhibitors. In the case of DHR3, it is experimentally difficult to address this question as the ligand for DHR3 is unknown and we are scoring for a genetic endpoint resulting from events induced much earlier in larval development. It has been demonstrated that vitamin D3 [63], [64] and all-*trans*-retinoic acid [65] both induce activation of S6K1 within minutes of administration to cells. Moreover, in the case of vitamin D3, it was shown that these effects were mediated through protein phosphatases PP1 and PP2A in a vitamin D3 receptor (VDR)-dependent manner. VDR appears to directly interact with the catalytic subunits of PPI and PP2A, and vitamin D3 acts to disrupt this interaction and enhance an interaction between VDR and S6K1, stabilizing S6K1 in its phosphorylated active state [63], [64]. However, depleting DHR3 levels by RNA interference blunts both dS6K T398 and d4E-BP T37/T46 phosphorylation, suggesting that DHR3 acts upstream or at the level of dTORC1. Identification of potential partners for DHR3-PS may be useful in determining, at the molecular level, the mechanism by which DHR3 controls cell growth and dS6K activity.

The data further support the notion that a ligand exists for DHR3, and that the ligand is required for many of the pleiotropic activities of DHR3. Those NRs that bind steroid hormones are, in general, high-affinity receptors, whereas the low-affinity NRs bind ligands that are present in high concentration, such as dietary nutrients [66]. The observation that an NR, generated by fusing the DHR3 LBD with the DBD of Gal4, is transcriptionally active in a number of specific embryonic and larval tissues suggests that such a ligand is widely present [67]. Given the role of dTOR/dS6K as a nutritional effector [14], it is interesting to note that the chimeric DHR3/Gal4 NR is active in organs that provide basal nutrients, in particular, in a group of cells of the larval midgut, which are essential for the transfer of nutrients to the hemolymph [67]. Importantly, the mammalian orthologues to DHR3 and its partner E75 are retinoid-related orphan receptor (ROR)α and Rev-erb (NR1D)α, respectively [68]. As in *Drosophila*, the NR1D subgroup functions as dominant transcriptional silencers by inhibiting transactivation mediated by RORα [68]. Interestingly, it was recently reported that RORα-deficient mice, like S6K1-deficient mice [69], exhibit reduced fat-pad mass, smaller adipocytes, and resistance to diet-induced obesity [70]. Moreover, in solving the X-ray structure of the RORα LBD, it was revealed that cholesterol was bound in the ligand-binding pocket [71]. While the *Drosophila* NR, DHR96, has recently been shown to bind cholesterol thereby modulating cholesterol homeostasis [72], this does not exclude the possibility that DHR3 could also bind cholesterol. However, the predicted models of the structure of DHR3 indicate that the size of the ligand-binding pocket is smaller than those of either RORα or RORβ [73]. Given the role of the mTOR/S6K1 nutrient-responsive pathway in mammals [74], it raises the possibility that DHR3 is a low-affinity receptor for an abundant nutrient ligand. Identification of this specific ligand constitutes the next issue to investigate.

## Materials and Methods

### Fly stocks and genetics

The following fly strains were used: *dS6K^l1^* and UAS-dS6K; ap-Gal4 [18]; UAS-Tsc1/2 [7]; UAS-PTEN [75]; pumpless-Gal4 [14]; *DHR3^G60S^* and *DHR3^R107G^* [51]; eyeless-Gal4 [76]; Cre-lox (a generous gift from K. Basler); actin5c>CD2>Gal4,UAS-GFP [77]; and da-Gal4, actin-Gal4, engrailed-Gal4,UAS-GFP, *FRT-42D,M(2)53*, and *FRT-42D,P(y+)44B* (Bloomington stock center). Because *y+* and *w+* markers were used, all the experiments were performed in a *y,w* genetic background. In the screen, lines with about 5000 independent EPy+ insertions [39] were mated to ap-Gal4>UAS-dS6K virgin females and offspring were scored for modulation of the bent-down wing phenotype. Approximately 900 suppressor and 100 enhancer lines were further analyzed for their effects on wing development when mated to ap-Gal4 virgin females. In a third step, 90 enhancer and 130 suppressor lines were retained and mated to either ap-Gal4>UAS-dS6K or ap-Gal4>UAS-S6K1^dE/D3E^ virgin females, to test their differential effect on dS6K versus S6K1^dE/D3E^. For the EMS revertant screen, about 500 DHR3-R-EP males were starved overnight and then transferred on wet paper containing a 25 mM EMS solution in 10 mg/ml sucrose. After one day, these males were mated to approximately 1500 ap-Gal4>UAS-dS6K virgin females. Flies were then transferred every day for egg laying. An estimated 150,000 F1 flies were obtained; as both parental lines were balanced over a CyO chromosome, about 50,000 flies were screened for the reversion of the bent-down-wing phenotype.

### Mapping and cDNA constructs

Localization of the EP insertions was performed as described [39]. To generate UAS-DHR3-RNAi, a PCR fragment spanning the DHR3 reading frame from Leu^114^ to Lys^265^ was cloned as described [78]. Congruent results were obtained by repeating the experiments with 2 other distinct UAS-DHR3-RNAi strains provided by H. Tricoire and the National Institute of Genetics (http://www.nig.ac.jp/). For RACE-PCR, polyA+ cDNAs were obtained by using the RNeasy kit and Oligotex mRNA purification (both from Qiagen) and then amplified with the SMART RACE cDNA Amplification Kit (Clontech). 5′ RACE to obtain endogenous cDNAs and the chimeric DHR3-EP cDNAs followed a 2-step process: first, using a DHRS-RR–specific primer (catggtctgctgtggcgtcacggaggc) and universal primer mix, and then by nested PCR using a combination of nested universal primer mix/DHRS-RR–specific primer (cggttgcgattaacacggtccaccac). UAS-S6K1^dE/D3E^ and DHR3 cDNAs were cloned in the pUAST vector and injected as previously described [18]. The RA-cDNA was kindly provided by Carl Thummel; the RB- cDNA was obtained from DGRC; the RS transcript was artificially generated by truncation of the RA-cDNA lacking the AUG initiator codon upstream of the DBD coding sequences. To identify EMS point mutations, DHR3 coding sequences were PCR amplified from the genomic DNA of revertant flies. Fragments were then sequenced and searched for double picks, as compared with wild-type genomic DNA. Identified point mutations were confirmed by independent repetition of the entire procedure.

### Tissue analyses

Larval tissues were dissected, stained as previously described [18], and then observed on a Leica Sp2 confocal microscope. For SEM, flies were fixed by successive baths of increasingly concentrated ethanol solution, up to 90%, and directly observed on an S-3000N HITACHI scanning-electron microscope. To measure eye area, *eye-flp*;*FRT-42D,M(2)53* females were mated to *FRT-42D,P(y+)44B* control males, and to *FRT-DHR3^K243X^* and *FRT-DHR3^W284X^* mutant males. Photographs of offspring female flies were used to measure the area of homozygous eyes and heterozygous thoraces, as described [18]. To circumvent potential individual variation, the eye size of each individual was normalized to its corresponding thorax. The ommatidia size was measured from SEM pictures of 6 flies of identical size for each genotype.

### Biochemical and immunohistochemical analyses

Protein extracts were prepared and western blotting was performed as previously described [13]. To select prepupae, wandering larvae of the corresponding phenotype were collected and transferred to a new tube. After 8 hours, newly formed prepupae and late third-instar larvae were collected to make protein extracts. The in vitro dS6K kinase activity assays were performed on second-instar larval extracts, essentially as described [13] using histone H2B as the substrate [79]. The antiserum to DHR3 was produced commercially by Eurogentec. The peptides ^144^QMRAQSDAAPDSSYYD^159^ and ^209^SADYVDSTTYEPRSTI^224^ were used to immunize rabbits. The specific anti-peptide antibodies were then affinity purified as previously described [80].

## Supporting Information

Figure S1 EP-elements and dS6K interactions at the dorsal wing compartment. The *ap* promoter directed Gal4 expression within the dorsal compartment of the wing imaginal disc (A) to induce UAS-dS6K (B–D) with various UAS constructs: (F) EP-12.190 induces a bending-up of the wing acting along the antero-posterior axis; (G) EP-21.118 induces a bending-down of the wing following the antero-posterior axis. Dorsal side is to the left and ventral is to the right in each wing photograph.
(1.93 MB TIF)

Figure S2 DHR3 transcripts and polypeptides. (A) The EP12.218 (E1) and EP23.014 (E2) are inserted into chromosome 2R at nucleotides 6107302 and 6107230 respectively. (B) 5′end of a chimeric mRNA produced upon EP induction by Gal4; EP sequences are italicized. The following DHR3-specific primers were used for the first step (catggtctgctgtggcgtcacggaggc) and for the nested step (cggttgcgattaacacggtccaccac). (C) 5′end of a novel DHR3 transcript (DHR3-RS), starting at nucleotide 6097546 of chromosome 2R. The nucleotide sequence corresponding to the classically referenced DHR3 2nd exon is shown in normal characters; the first initiator codon for each transcript is boxed. (D) EMS point mutations (boxed letters) of the DHR3 polypeptide PA; G60S (G) and R107G (R) affect the DBD, whereas K243X (K) and W284X (W) are early stop codons within the LBD (underlined). Methionines are shown in bold; the peptides 144QMRAQSDAAPDSSYYD159 and 209SADYVDSTTYEPRSTI224, which were used for rabbit immunizations, are highlighted.
(0.02 MB PDF)

Figure S3 Transheterozygous DHR3-EP have reduced prepupal weight. Heterozygous control (Co) and transheterozygous DHR3-EP (EP) wandering larvae were collected. Males and females were transferred in separate tubes. Weights were then determined on 20 prepupae formed after 8 hours for each sample. As compared to control, transheterozygous DHR3-EP exhibit a significant 8% reduction in body weight (**P<0.01).
(0.85 MB TIF)

Figure S4 DHR3 RNAi blunts DHR3 mRNA levels and the phosphorylation of dS6K and d4E-BP. (A) Q-PCR from either act-Gal4 (Co) or act-Gal>DHR3-RNAi (Ri) white prepupa. (B–E) Western-blot analysis of Drosophila α-Tubulin levels (B), dS6K levels (C), and the phosphorylation of dS6K T398 (D) and d4E-BP T37/T46 (E) in either act-Gal4 (Co) or act-Gal>DHR3-RNAi (Ri) third instar larval extracts.
(1.71 MB TIF)

Table S1 Screening for modulators of dS6K.
(0.10 MB PDF)

Table S2 Complementation tests between DHR3 mutants.
(0.03 MB PDF)
