# Supplementary figures and images for: The Nuclear Receptor DHR3 Modulates dS6 Kinase–Dependent Growth in Drosophila
Source: PLoS Genet. 2010 May 6;6(5):e1000937. doi: 10.1371/journal.pgen.1000937 (PMC2865512; doi:10.1371/journal.pgen.1000937)

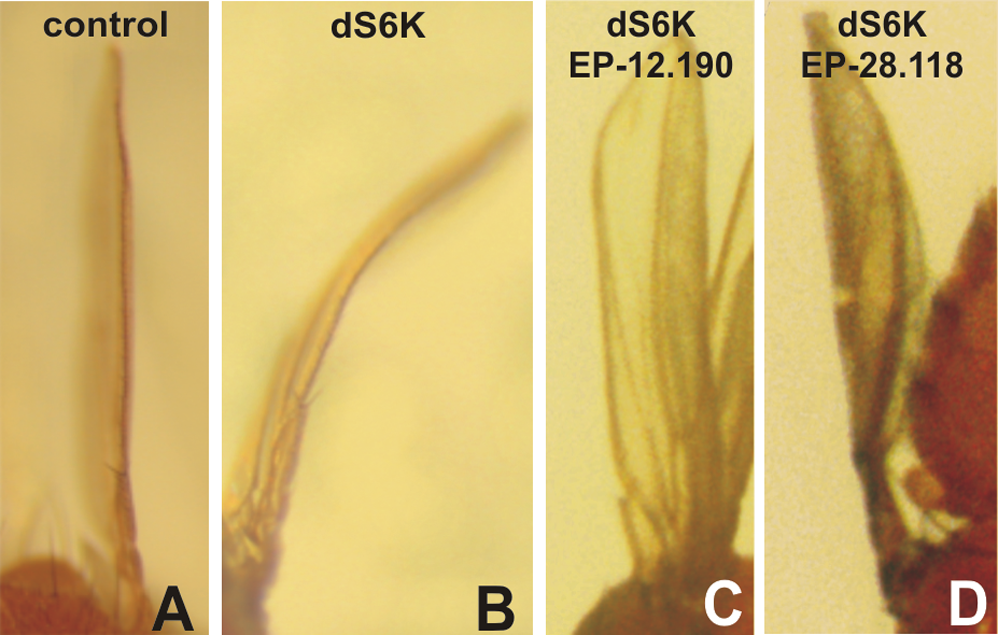

Supplement: Figure S1 — EP-elements and dS6K interactions at the dorsal wing compartment. The ap promoter directed Gal4 expression within the dorsal compartment of the wing imaginal disc (A) to induce UAS-dS6K (B–D) with various UAS constructs: (F) EP-12.190 induces a bending-up of the wing acting along the antero-posterior axis; (G) EP-21.118 induces a bending-down of the wing following the antero-posterior axis. Dorsal side is to the left and ventral is to the right in each wing photograph. (1.93 MB TIF) [file pgen.1000937.s001.tif]

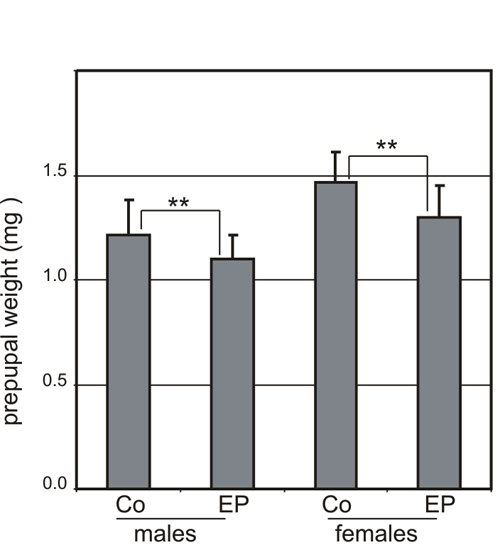

Supplement: Figure S3 — Transheterozygous DHR3-EP have reduced prepupal weight. Heterozygous control (Co) and transheterozygous DHR3-EP (EP) wandering larvae were collected. Males and females were transferred in separate tubes. Weights were then determined on 20 prepupae formed after 8 hours for each sample. As compared to control, transheterozygous DHR3-EP exhibit a significant 8% reduction in body weight (**P<0.01). (0.85 MB TIF) [file pgen.1000937.s003.tif]

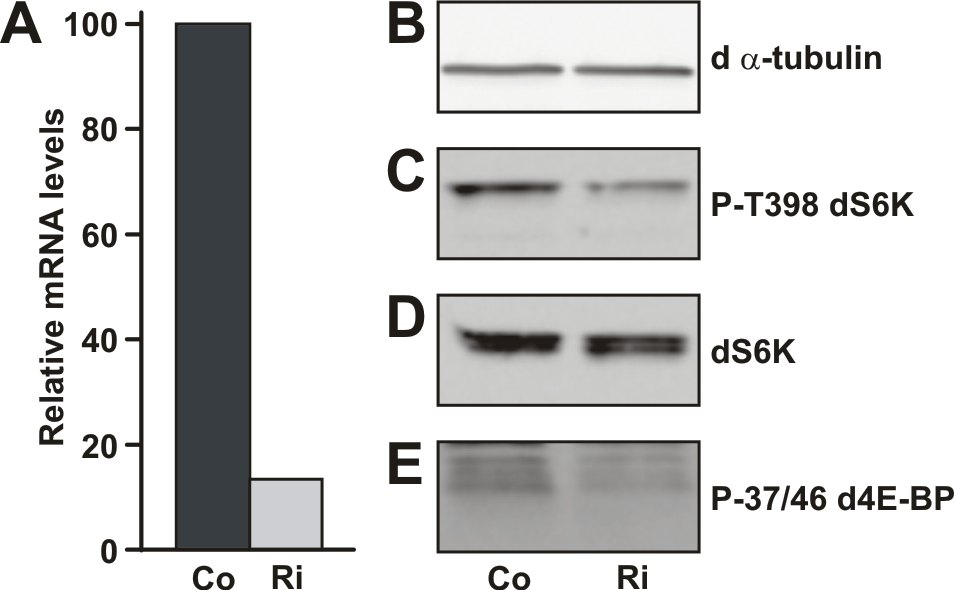

Supplement: Figure S4 — DHR3 RNAi blunts DHR3 mRNA levels and the phosphorylation of dS6K and d4E-BP. (A) Q-PCR from either act-Gal4 (Co) or act-Gal>DHR3-RNAi (Ri) white prepupa. (B–E) Western-blot analysis of Drosophila α-Tubulin levels (B), dS6K levels (C), and the phosphorylation of dS6K T398 (D) and d4E-BP T37/T46 (E) in either act-Gal4 (Co) or act-Gal>DHR3-RNAi (Ri) third instar larval extracts. (1.71 MB TIF) [file pgen.1000937.s004.tif]
