# Supplementary material for: The Nuclear Receptor DHR3 Modulates dS6 Kinase–Dependent Growth in Drosophila
Source: PLoS Genet. 2010 May 6;6(5):e1000937. doi: 10.1371/journal.pgen.1000937 (PMC2865512; doi:10.1371/journal.pgen.1000937)
Supplement: Figure S2 — DHR3 transcripts and polypeptides. (A) The EP12.218 (E1) and EP23.014 (E2) are inserted into chromosome 2R at nucleotides 6107302 and 6107230 respectively. (B) 5′end of a chimeric mRNA produced upon EP induction by Gal4; EP sequences are italicized. The following DHR3-specific primers were used for the first step (catggtctgctgtggcgtcacggaggc) and for the nested step (cggttgcgattaacacggtccaccac). (C) 5′end of a novel DHR3 transcript (DHR3-RS), starting at nucleotide 6097546 of chromosome 2R. The nucleotide sequence corresponding to the classically referenced DHR3 2nd exon is shown in normal characters; the first initiator codon for each transcript is boxed. (D) EMS point mutations (boxed letters) of the DHR3 polypeptide PA; G60S (G) and R107G (R) affect the DBD, whereas K243X (K) and W284X (W) are early stop codons within the LBD (underlined). Methionines are shown in bold; the peptides 144QMRAQSDAAPDSSYYD159 and 209SADYVDSTTYEPRSTI224, which were used for rabbit immunizations, are highlighted. (0.02 MB PDF) [file pgen.1000937.s002.pdf]

A

cacacacgcactttccgatagagcaaatg**E1**gggaagagcgagcgctggcgggagtggg
agagcgggagagagtgcgtgagagtggagcgttcgttgactgtta**E2**tgcgggagcgtg

B *AATTCAATTCAAACAAGCAAAGTGAACACGTCGCTAAGCGAAAGCTAAGCAAATA*
AACAAGCGCAGCTGAACAAGCTAAACAATCTGCAGTAAAGTGCAAGTTAAAGTGAATCAA
*TTAAAAGTAACCAGCAACCAAGTAAATCAACTGCAACTACTGAAATCTGCCAAGAA*CTCA
AATTGAGATAATTCCATGCAAAGTCTGCGGCGACAAGTCATCCGGCGTGCATTACGGAGT
GATCACCTGCGAGGGCTGCAAGGGATTCTTTCGAAGATCGCAAAGCTCCGTGGTCAACTA
CCAGTGTCCGCGCAACAAGCAATGTGTGGTGGACCGTGTTAATCGCAACCGATGTCAATA
TTGTAGACTGCAAAAGTGCCTAAAACTGGGA**ATG**AGCCGTGATG..........
M S R D . . . .

C TCACCTGCGAGGGCTGCAAGGGATTCTTTCGAAGATCGCAGAGCTCCGTGGTCAA
CTACCAGTGTCCGCGCAACAAGCAATGTGTGGTGGACCGTGTTAATCGCAACCGATGTCA
ATATTGTAGACTGCAAAAGTGCCTAAAACTGGGA**ATG**AGCCGTGATG...........
M S R D . . . .

D

MYTQRMFDMWSSVTSKLEAHANNLGQSNVQSPAGQNNSSGSIKAQIEIIPCKVCGDKSS
GVHYGVITCEGCKGFFRRSQSSVVNYQCPRNKQCVVDRVNRNRCQYC**R**LQKCLKLG**M**SR
DAVKFGR**M**SKKQREKVEDEVRFHRAQ**M**RAQSDAAPDSSVYDTQTPSSSDQLHHNNYNSY
SGGYSNNEVGYGSPYGYSASVTPQQT**M**QYDISADYVDSTTYEPRSTIIDPEFISHADGD
INDVLI**K**TLAEAHANTNTKLEAVHDMFRKQPDVSRILYYKNLGQEEL**W**LDCAEKLTQMI
QNIIEFAKLIPGFMRLSQDDQILLLKTGSFELAIVRMSRLLDLSQNAVLYGDVMLPQEA
FYTSDSEEMRLVSRIFQTAKSIAELKLTETELALYQSLVLLWPERNGVRGNTEIQRLFN
LSMNAIRQELETNHAPLKGDVTVLDTLLNNIPNFRDISILHMESLSKFKLQHPNVVFPA
LYKELFSIDSQQDLT
