# Supplementary material for: The Nuclear Receptor DHR3 Modulates dS6 Kinase–Dependent Growth in Drosophila
Source: PLoS Genet. 2010 May 6;6(5):e1000937. doi: 10.1371/journal.pgen.1000937 (PMC2865512; doi:10.1371/journal.pgen.1000937)
Supplement: Table S1 — Screening for modulators of dS6K. (0.10 MB PDF) [file pgen.1000937.s005.pdf]

A

LINE	UAS-DS6K	ap-Gal4	UAS-dE/D3E	Chsome	Map	nucleotide	gene
14,020	A/P up - -	A/P up - - -	A/P up - -	X	2F	2211773	
25,179	TV down ++	(----)	TV down ++	X	3C	3057186	
25,198	TV down ++	TV (----)	TV down +	X	3C	3057186	
25,159	down +++	(----)	down ++	X	3F	3690485	
26,187	down +++	(----)	down ++	X	5E	6177714	
22,155	down ++++	down +	down +++	X	11B	12365975	rab 40
25,175	down +++	(----)	down ++	X	12E	14215238	
11,018	down +++	(----)	down +++	X	12F	14726697	
20,167	A/P down +++	A/P down ++	A/P down +++	X	18B	19154193	
12,002	down +++	(----)	down ++	X			
13,036	down +++	(----)	down +++	X			
18,250	down ++++	down +	down +++	X			
24,183	down +++	(----)	down +	X			
18,113	down +++	(----)	down ++	2	24E	4282935	
26,080	blister up - -	blister up - -	blister up - -	2	28A	7576629	
23,024	down ++++	(----)	down ++	2	28D	7991728	peste
28,133	down +++	(----)	down ++	2	28D	7991787	peste
16,219	addV down +++	addV	addV down +++	2	29A	8271542	
13,148	down +++	(----)	down ++	2	30D	9782358	
24,188	up - -	up - -	up - -	2	34C	13549293	
27,136	down ++++	(----)	down ++++	2	38C	20310996	spir
15,196	down +++	(----)	down +++	2	38C	20311312	spir
19,020	down ++++	(----)	down ++++	2	38C		spir
25,010	down +++	(----)	down ++	2	41F	1509876	
12,177	fold down +++	fold	fold down +++	2	44B	4119835	
28,122	fold down +++	fold+	fold down ++	2	44D	4558214	
13,116	up -	up - - - -	up -	2	45F	5440342	
23,014	down ++++	(----)	down +++	2	46F	6107230	DHR3
12,218	down ++++	(----)	down +++	2	46F	6107302	DHR3
25,138	up - - - -	up - - - -	up - - - -	2	47A	6366134	
8,195	down +++	(----)	down ++	2	47A	6414247	
26,092	addV down +++	addV +	addV down ++	2	47F	7339990	
16,217	A/P up - -	A/P up - - -	A/P up - -	2	49B	8474745	
11,124	fold down ++++	fold down ++	fold down ++++	2	50C	9894698	
17,074	A/P up - - - -	blister up - - - -	A/P up - - - -	2	50F	10155404	
12,190	A/P up - -	A/P up - - -	A/P up - -	2	54B	13307092	
27,116	A/P up - - - -	blister up - - - -	A/P up - - - -	2	54B	13307828	
23,174	A/P up - -	A/P up - - - -	A/P up - -	2	54B	13307943	
13,211	blister up -	blister up - -	blister up -	2	54B	13308389	
24,095	blister up -	A/P up - -	blister up -	2	54B	13308443	
12,205	fold down ++++	fold down ++	fold down ++++	2	54C	13345933	
20,100	down +++	(----)	down ++	2	56D	15245342	
9,100	fold down +++	fold down +	fold down +++	2	56D	15519538	
4,035	addV down +++	addV	addV down ++	2			
7,064	down +++	(----)	down ++	2			
10,118	fold down +++	fold down +	fold down +++	2			
11,051	A/P down ++	(----)	A/P down ++	2			
20,176	up -	up - -	up -	2			
26,015	fold down ++++	fold down ++++	fold down ++++	2			
26,057	down +++	(----)	down ++	2			
27,110	up - -	up - - - -	up - -	2			
15,239	down +++	down +	down +	3	61A	131411	PDK1
15,235	down ++++	down +	down ++	3	61A	131694	PDK1
26,020	down ++++	down +	down +++	3	61A	131712	PDK1
14,342	down ++++	down +	down ++	3	61A	131771	PDK1
22,068	down ++++	down +	down +++	3	61A	131771	PDK1
25,092	down ++++	down +	down ++	3	61A	131781	PDK1
21,024	down ++++	down +	down +++	3	61A	131783	PDK1
18,193	down ++++	down +	down +++	3	61A	131807	PDK1
5,045	down ++++	down +	down ++	3	61A	131885	PDK1
23,180	down ++++	down +	down +++	3	61A	131955	PDK1
28,118	A/P down ++	A/P down +	A/P down ++	3	61D	883054	
21,121	up -	up - -	up -	3	64C	4750797	
17,251	down +++	(----)	down ++	3	66A	7798582	
24,061	down ++++	down +	down +++	3	66E	8942100	CPEB
25,112	down +++	(----)	down +++	3	69A	12259860	
18,100	fold down ++++	fold +	fold down ++	3	71E	15586673	
22,048	A/P down ++++	A/P down +	A/P down ++++	3	76D	19889519	

**24,159**	fold down ++	fold down +++	fold down +++	**3**	**78C**	21233150	
**21,070**	down +++	down +	down ++	**3**	**79A**	21763733	
**10,045**	down ++++	down +	down ++++	**3**	**85B**	4723958	
**11,152**	down ++++	down ++++	down ++++	**3**	**89B**	12106222	
**24,034**	fold down +++	fold down ++	fold down +++	**3**	**89F**	12944822	
**26,125**	up - -	Up - - - -	up - -	**3**	**93B**	16890857	
**10,116**	down +++	(----)	down ++	**3**	**97D**	22702732	
**13,123**	down +++	(----)	down ++	**3**	**99A**	25081089	
**13,145**	down +++	down +	down ++	**3**	**99C**	25585909	
**26,094**	down +++	(----)	down ++	**3**	**99D**	25758758	
**17,025**	down ++	(----)	down +	**3**	**100F**	27677651	
**22,067**	down ++++	down +	down +++	**3**	**100F**	27761974	PTB
**26,139**	down +++	(----)	down +++	**3**	**100F**	27763278	PTB
**12,046**	down +++	(----)	down +	**3**	**100F**	27810394	PTB
**6,077**	down +++	(----)	down +	**3**	**100F**	27811458	PTB
**6,027**	down ++	(----)	down +	**3**			
**12,100**	up -	up - -	up -	**3**			
**12,163**	down +++	down +	down +++	**3**			
**16,030**	down +++	(----)	down +++	**3**			
**17,203**	up - - -	up - - - -	up - - -	**3**			
**19,082**	down +++	down +	down ++	**3**			
**19,100**	down ++++	(----)	down +++	**3**			
**20,131**	down ++++	(----)	down +++	**3**			
**22,105**	up -	up -	up -	**3**			
**23,129**	down +++	down +	down ++	**3**			
**27,161**	down +++	(----)	down +++	**3**			
**26,087**	down +++	(----)	down +++	**4**			

B

**Class**	**phenotype description**
down ++++	very strong bending down
down +++	strong bending down
down ++	intermediate bending down
down +	bending down as dS6k effect
(----)	flat wing, similar to wild type
Up **-**	wing bent up weakly
Up **- -**	wing bent up strongly
Up **- - - -**	extreme bent up of the wing
A/P	effect following antero-posterior axis (width effect)
TV	veins thicker
fold	wing folded
addV	additional veins
blister	default of wing blades apposition
@	cytogenic location mapped
	irrelevant for dS6K
	effect on wing differentiation
	suppressor
	antero/posterior effect
	similar enhancer with either kinase
	enhancer, greater with dS6K than active-mS6K
	Strong enhancer

Table S1. Screening for modulators of dS6K. (A) The 95 EP lines finally retained are listed. Column legend: **line**, EP number; **UAS-dS6K**, phenotype when co-induced with dS6K; **ap-Gal4**, phenotype when induced alone; **UAS-dE/D3E**, phenotype when co-induced with the active mammalian S6K; **Chsome**, chromosomal location; **Map**, cytological location; **nucleotide**, genomic location according to the flybase consortium; **gene**, potential candidate. (B) Description of the phenotypes indicated in A and of classes of enhancers or suppressors regarding the color usage.
