# Supplementary material for: The Nuclear Receptor DHR3 Modulates dS6 Kinase–Dependent Growth in Drosophila
Source: PLoS Genet. 2010 May 6;6(5):e1000937. doi: 10.1371/journal.pgen.1000937 (PMC2865512; doi:10.1371/journal.pgen.1000937)
Supplement: Table S2 — Complementation tests between DHR3 mutants. (0.03 MB PDF) [file pgen.1000937.s006.pdf]

	EP12.218	*EP23.014*	*K10308*	*G60S*	*R107G*	*K247X*	*W288X*
EP12.218	***1/2***	***1/2***	***L3***	***L3***	***L3***	***L3***	***L3***
EP23.014		***1/2***	***L3***	***L3***	***L3***	***L3***	***L3***
K10308			***Em***	***Em***	***Em***	***Em***	***Em***
G60S				***Em***	***Em***	***Em***	***Em***
R107G					***Em***	***Em***	***Em***
K247X						***Em-L2***	***Em-L2***
W288X							***Em-L2***

Table S2: Complementation tests between DHR3 mutants. EP12.218 and EP23.014 are the DHR3-EP elements selected in the screen. K10308 is a P-element located at nucleotide 2R:6098599 close to the second DHR3 exon. G60S and R107G are previously described DHR3 mutants affecting the DBD, whereas K247X and W288X are mutants in the LBD obtained in the EMS revertant screen (see text).

The various mutant trans-combinations are semi-lethal (1/2), or lethal at embryogenesis (Em) or at third-instar larval stage (L3). Most of the LBD mutant combinations led to embryonic lethality, although it was possible to find a very few survivors up to the second-instar larval stage (Em-L2).
